# Adipose-derived mesenchymal stem cells promote the malignant phenotype of cervical cancer

**DOI:** 10.1038/s41598-020-69907-x

**Published:** 2020-08-26

**Authors:** Rosario Castro-Oropeza, Karla Vazquez-Santillan, Claudia Díaz-Gastelum, Jorge Melendez-Zajgla, Cecilia Zampedri, Eduardo Ferat-Osorio, Arturo Rodríguez-González, Lourdes Arriaga-Pizano, Vilma Maldonado

**Affiliations:** 1 Epigenetics Laboratories, National Institute of Genomic Medicine (INMEGEN), 14610 Mexico City, Mexico; 2 Functional Genomics Laboratories, National Institute of Genomic Medicine (INMEGEN), 14610 Mexico City, Mexico; 3 Gastrosurgery Service, UMAE, National Medical Center “Siglo XXI”, Mexican Institute of Social Security (IMSS), Mexico City, Mexico; 4 Medical Research Unit on Immunochemistry, National Medical Center “Siglo XXI”, Mexican Institute of Social Security (IMSS), Mexico City, Mexico

**Keywords:** Cancer microenvironment, Cancer microenvironment, Growth factor signalling, Growth factor signalling

## Abstract

Epidemiological studies indicate that obesity negatively affects the progression and treatment of cervical-uterine cancer. Recent evidence shows that a subpopulation of adipose-derived stem cells can alter cancer properties. In the present project, we described for the first time the impact of adipose-derived stem cells over the malignant behavior of cervical cancer cells. The transcriptome of cancer cells cultured in the presence of stem cells was analyzed using RNA-seq. Changes in gene expression were validated using digital-PCR. Bioinformatics tools were used to identify the main transduction pathways disrupted in cancer cells due to the presence of stem cells. In vitro and in vivo assays were conducted to validate cellular and molecular processes altered in cervical cancer cells owing to stem cells. Our results show that the expression of 95 RNAs was altered in cancer cells as a result of adipose-derived stem cells. Experimental assays indicate that stem cells provoke an increment in migration, invasion, angiogenesis, and tumorigenesis of cancer cells; however, no alterations were found in proliferation. Bioinformatics and experimental analyses demonstrated that the NF-kappa B signaling pathway is enriched in cancer cells due to the influence of adipose-derived stem cells. Interestingly, the tumor cells shift their epithelial to a mesenchymal morphology, which was reflected by the increased expression of specific mesenchymal markers. In addition, stem cells also promote a stemness phenotype in the cervical cancer cells. In conclusion, our results suggest that adipose-derived stem cells induce cervical cancer cells to acquire malignant features where NF-kappa B plays a key role.

## Introduction

The accumulation of mutations, genetic predisposition of some factors, exposure to environmental agents, and lifestyle all play important roles in the carcinogenic process. Recent studies have revealed that obesity complicates the clinical course of cancer^1–3^. Obesity is defined as body mass index (BMI) over 30 kg/m^2^ and is characterized by excessive accumulation of adipose tissue in the body. Interestingly, the International Agency for Research on Cancer (IARC) reported that body fatness increases cancer risk in 13 cancer types denominated obesity-related cancers (ORC), among them, cervical cancer (CC)^4,5^. Further supporting this association, Calle et al. studied approximately 90,000 women and reported that excess BMI is associated with an increased risk of invasive cancer^6^, with cervical cancer being one of the most affected by obesity, thus increasing the relative risk of death 3.2 times more in patients with BMI > 35^6–8^. The relationship between obesity and cancer may be explained by many factors, including altered hormonal signaling, chronic inflammation, fatty acid metabolism, abnormal regulation of insulin and other diet compounds involved in adipose tissue metabolism^9–11^.

Adipose tissue contains multipotent stem cells that are able to self-renew and differentiate into multiple cell lineages^12^. This cell population, referred to as ADSCs (adipose tissue-derived stem cells), has a crucial role in cancer^13–15^. ADSCs can be chemoattracted to solid tumors and secrete cytokines, including IL6 and vascular endothelial growth factor (VEGF), which promote the inflammatory microenvironment and influence tumorigenesis^16–20^. ADSCs could transform the behavior of tumor cells to make them more aggressive^21,22^, able to survive, proliferate^23,24^, migrate and seed new tumors. Recently, it has been proposed that the angiogenic and growth factors secreted by ADSCs greatly contribute to facilitating the access of nutrients and oxygen, allowing rapid tumor growth^18^. The molecular mechanism of the interaction between tumor cells and ADSCs is still unknown, so increased efforts are needed to understand how ADSCs contribute to tumor malignancy.

NF-kappa B is a family of transcription factors that regulate multiple genes involved in immune response, cell survival, proliferation, angiogenesis, and metastasis^25–27^. NF-kappa B signaling can be activated by the canonical pathway mainly characterized by the translocation of p50: p65 dimer or by the noncanonical pathway characterized by the translocation of p52: RelB dimer^28,29^. Notably, the NF-kappa B pathway is altered in obesity^30,31^ and cancer pathologies^26,27,32–35^. In this study, we analyzed the biological impact and molecular mechanisms of ADSCs in CC cells that are still unknown. We cocultured HeLa cells with ADSCs, evaluated their transcriptome and performed in vitro and in vivo assays to reveal the influence of ADSCs and the molecular mechanisms that alter the phenotype of CC cells.

Our results showed that ADSCs promote cell movement, angiogenesis, migration, and the epithelial–mesenchymal transition (EMT) and increase the malignant properties of CC cells through the positive regulation of NF-kappa B signaling, a pathway involved in initiation, progression and resistance to treatment in various types of cancer.

## Materials and methods

### Cell culture

HeLa, SiHa, CaSki, HaCaT and ADSC were obtained from ATCC (Manassas, VA, USA) and cultured in DMEM medium supplied with 5% fetal bovine serum (FBS) (ATCC, 30-2020) at 37 °C/5% CO_2_. To grow and expand ADSCs we used a commercially available medium (Mesenchymal Stem Cell Basal Medium, ATCC PCS 500030) containing essential and non-essential amino acids, vitamins, other organic compounds, trace minerals, and inorganic salts. This medium was supplemented with a specific growth kit for ADSC (ATCC No. PCS­500­040) containing the following growth supplements: (low serum (2% FBS), FGF basic, EGF and l­alanyl­l­glutamine).

### Isolation, culture and characterization of ADSCs

Adipose tissue samples were obtained from 3 female cancer-free patients undergoing gastric bypass, with BMI > 40. The samples were collected from Specialty Hospital, XXI Century National Medical Center of the Mexican Social Security Institute (IMSS). This study was conducted according to institutional guidelines under an approved protocol by the ethics committee of Comite Local de Investigación del Hospital de Especialidades “Dr. Bernardo Sepúlveda Gutiérrez” del Centro Médico Nacional Siglo XXI del Instituto Mexicano del Seguro Social. All donors provided written informed consent.

The enriched ADSC population was isolated from excised human adipose tissue as previously published^36,37^. Briefly, freshly tissues were washed with PBS and minced into small pieces and samples were incubated with collagenase I at 0.075% (SCR103, Millipore USA MA) for 40 min at 37 °C with gently shaker. After centrifugation, the stromal fraction containing the ADSC was collected and seeded with DMEM medium supplemented with streptomycin/penicillin 1X (30-2300 ATCC, Virginia, USA) and 5% FBS. ADSCs were expanded in DMEM-5% FBS.

The identity of the ADSCs, the expression of stromal markers (CD44 (MACS Miltenyi Biotec 130-095-195, CA, USA), (CD90 Millipore FCMAB211F, MA, USA) were analyzed by Flow Cytometry. Also, we analyzed the absence of hematopoietic marker (CD31, CBL468F Millipore, MA, USA), and endothelial marker (CD45 FCMAB118F Millipore, MA, USA). The mouse IgG1-PE (103.092-212 MACS Miltenyi Biotec) and Mouse IgG-FIT (130-092-213 MACS Miltenyi Biotec) were used how isotype control antibodies.

### HeLa-ADSC coculture assays

Indirect coculture assays were used in order to guaranty the isolation of the two cell lines through a permeable membrane, but also to kept them in the same microenvironment. HeLa cells (950,000) were seeded in the upper compartment of a Transwell system (#3420 Corning Costar, NY, USA) with a pore size of 3.0 μm. Subsequently, 450,000 ADSCs obtained from patients or from the ATCC were seeded onto the lower compartment. The coculture remained for 24 h in serum-free DMEM.

### Production of conditioned medium

*Coculture conditioned medium (Coculture-CM).* Serum-free DMEM was harvested from the 24 h HeLa-ADSC coculture. *ADSC conditioned medium (ADSC-CM)* ADSCs (450,000) were seeded and cultured with serum-free DMEM for 24 h. *HeLa conditioned medium (HeLa-CM)* HeLa cells (950,000) were seeded and cultured with serum-free DMEM for 24 h. All generated media were filtered to eliminate any cells.

### RNA sequencing

Total RNA was isolated from HeLa and HeLa cells co-cultured with ADSC (N = 3) using QIAzol (79306, QIAGEN, MD, USA). RNA concentration and integrity were evaluated using a Bioanalyzer, only samples with an RNA integrity number (RIN) greater than 9 were considered for subsequent analysis. HeLa cells cultured in the presence or absence of ADSC was subjected to RNAseq analysis by Illumin platform (GAII). Three biological replicates were used for the analysis and 20 millions of “reads” per replicate were obtained approximately. Sequencing data were analyzed with CLC Genomics workbench (7CLC BioCambridge), and differential expression was determined between groups using the EdgeR algorithm. Only genes with a fold change increment higher than 2 or less than − 2, a p-value ≤ 0.05, and adjusted p-values (FDR) ≤ 0.1 were further considered for subsequent analysis. In order to validate RNAseq data we elected DE genes with mid to high read counts since the variance in those data is less and the differences are more reliable. We also check the expression values of those transcripts across replicates and we choose genes with constant read counts between replicates. Finally, we use databases such as IPA and Metacore to dissect the functional interpretations of DE genes and select candidate genes according to its relevance in cancer development and progression. Elected genes were analyzed by Digital PCR.

### Gene set enrichment analysis (GSEA)

We imported the data obtained from our RNAseq to the GSEA software downloaded from the website: https://software.broadinstitute.org/gsea/index.jsp. The sets of genes related to different gene ontology processes served as reference genes to determine the biological processes enriched in our data. We only consider gene set enrichment dataset having a false discovery rate (FDR) < 0.25 and a normalized enrichment score (NES) > 1.2.

### Key pathway analysis

Ingenuity Pathway Analysis software (IPA-QIAGEN), Metacore software and Key Pathway Advisor were used to identify the main biological processes altered by the presence of ADSC in HeLa, as well as to infer which genes are involved in the regulation of essential cellular pathways. Those tools use the list of differentially expressed.

### Overall survival analysis

Overall survival of CC patients was analyzed in the website: https://kmplot.com/analysis/. The software simultaneously integrates gene expression and clinical data. We used the Pan-cancer RNA-seq section and analyze only cervical squamous cell carcinoma to generate each Kaplan–Meier survival graph, then, we calculated the risk ratio with a 95% confidence interval and the p value of logarithmic range.

### Droplet digital PCR

All ddPCR assays were performed using the QX200 digital drop PCR system according to the manufacturer's instructions (Bio-Rad)^38^.

Briefly, each reaction of EvaGreen ddPCR Supermix (#1864034) including the specific primers and cDNA was emulsified with oil (#1864006) and fractionated up to 20,000 drops in the QX200 generator. The droplets were transferred to a 96-well plate (#10023379) to carry out a PCR amplification following these conditions: 1 × (95 °C for 5 min), 40 × (95 °C for 30 s, Tm °C for 30 s, 72 °C for 30 s), 1 × (4 °C for 5 min, 90 °C for 5 min), 10 °C ∞. The positive drops containing at least one copy of amplifiable cDNA, exhibited an increase in fluorescence compared to negative drops. Fluorescent drops were quantified with the QX200 Droplet reader detector. Data analysis was performed in the QuantaSoft software and absolute expression values in copy number per μl were calculated using statistics for a Poisson distribution. Results are represented in copy number per μl of amplifiable cDNA.

### In vitro cell migration and invasion assays

To evaluate cell migration and invasion capacity of CC cells cultured in the presence of different chemoattractants (ADSC, NH3T3 cells, different CM, 5% FBS or serum free medium).

Cells (35,000) were seeded in the upper chamber and cultured in serum free medium, subsequently, 600 μl of medium with different chemoattractants were added. Cells were cultured at 37 °C and 5% CO_2_ for 24 h. The inserts were then removed from wells and cells on the upper surface of the transwell membrane were removed. Migrating cells located on the lower surface were rinsed with PBS and fixed with 4% paraformaldehyde (PFA). Finally, cells were stained with 0.1% crystal violet and images captured in a stereomicroscope were used to calculate migrating cells using the ImageJ software.

For the invasion assays, transwells covered with 50 μl of Matrigel were used. A total of 35,000 cells were seeded with serum-free medium in the upper chamber and subsequently, 600 μl of the corresponding chemoattractants were placed in the lower chamber. All conditions were carried out in the same way as the migration assays. At least three independent replicates and three technical replicates were carried out for each culture condition.

### Cell proliferation assays

Cell proliferation of HeLa cells was evaluated by the MTS method under two experimental conditions: (A) HeLa vs. HeLa co-cultured with ADSC. (B) HeLa in the presence of different medium (serum-free DMEM, DMEM supplemented with 5% FBS, conditioned medium of ADSC, conditioned medium of HeLa and medium obtained from HeLa-ADSC co-culture).

Briefly, 7,000 cells were seeded in triplicates in 96-well plates and incubated at 37° C in a 5% CO_2_ atmosphere in serum-free DMEM for condition "A" and in the correspondent medium for condition "B". Cell proliferation was evaluated at 0, 24 h, 48 h, 72 h, 100 h and 124 h and cells were incubated with the MTS reagent for 1 h. Late, cell proliferation was quantified using the CellTiter-96 non-radioactive cell proliferation assay kit (#G4000, Promega). Absorbance was measured at an optical density of 590 nm using a multidetector reader (Bekman Coulter).

### Cell cycle

Cell cycle assays were performed by flow cytometry using the DNA Reagent Kit (Cycletest Plus #340242, BD, Billerica, MA) following the manufacturer’s recommendations.

### Immunofluorescence assays

HeLa cells were grown on glass coverslips in 6-well plates and exposed to conditioned medium obtained from ADSC or serum-free medium during 24 h. Cells were then rinsed with 1X PBS and fixed with 4% paraformaldehyde (Sigma-Aldrich, St. Louis, MO, USA) for 30 min. Subsequently, cells were blocked with PBS and 5% bovine serum albumin (Sigma-Aldrich) for 2 h and then cells were incubated overnight in a humid chamber with the corresponding primary antibody: RelB, p65, p52, Vimentin, Fibronectin, N-cadherin or E-cadherin (Thermo-Fisher). The cells were washed and incubated with a corresponding secondary antibody (#W4028 or #W4018 Promega) for 1 h. Finally, cells were washed with PBS and the slides were mounted in Everbrite mounting medium with DAPI (Biotium Inc., Hayward, CA, USA) and stored at 4 °C. The fluorescence analysis was performed in a confocal microscope (Zeiss LSM 510).

### Zebrafish husbandry and xenotransplant assays

Zebrafish (Danio rerio) were maintained at a temperature of 28.5 °C on a pH of 7.4 with a 14 h. on and 10 h. off light cycle. We use a Tab Wik genetic background (provided by Dr. Ernesto Maldonado from ICMyL-UNAM and Dr. Francisco Carmona from IFC-UNAM), and we also use a zebrafish model (Tg(fli1a:EGFP)) harboring GFP expression in blood vessels (donated by Dr. Fernando López-Casillas from IFC-UNAM).

Zebrafish xenotransplants assays were approved by ethic Committee of INMEGEN. Animal care and husbandry followed international ethics standards.

For xenotransplant experiments, two days post-fertilization (dpf) zebrafish embryos were dechorionated and anesthetized with tricaine (MS-222; Sigma), then, cells were microinjected into the embryonic yolk sac region. To analyze the tumorigenic ability, 300 SiHa cells were injected in combination with 50, 100, 150 or 300 either ADSC, NIH3T3 or HaCaT cells into the yolk of Tab-wik zebrafish embryos. The microinjection protocol was carried out as previously reported^39^. For the migration assays, SiHa cells were transfected using Xfect (Clontech) and 5 μg of plasmid pGFP-R-VS or stained with a red PKH26-GL dye. Embryos were analyzed at 12 hpi (hours post injection) using an epifluorescence microscope (Zeiss). Four dpi (days post injection), larvae were analyzed by stereoscope to evaluate tumor formation.

### Extreme limiting dilution assays (ELDA)

In vivo limiting dilution assays were analyzed using the ELDA software. This model is focused on the estimation of the stem cell frequency across multiple data sets^40^. ELDA analysis considers the number of individuals who failed to form a tumor when limited cell dilutions are employed. The generated graph represents the slopes of the active cells logarithmic fraction with a 95% confidence interval.

### Angiogenesis

The zebrafish model Tg (fli1a: EGFP) was employed to inoculate SiHa cancer cells marked with a red PKH26-GL dye. The migration was evaluated at 12 hpi, and the formation of new blood cells was evaluated at 12 h and 3 days after the injection.

### Statistical analysis

At least 3 biological replicates of each experiment were made. Data were analyzed with the GraphPad Prism 5 program, in which Student’s t test or ANOVA was performed as appropriate, considering a p value of < 0.05 as statistically significant. The graphs show the average of the three replicates and the standard deviation (± SD).

## Results

### Isolation and characterization of ADSCs

**Figure 1:**
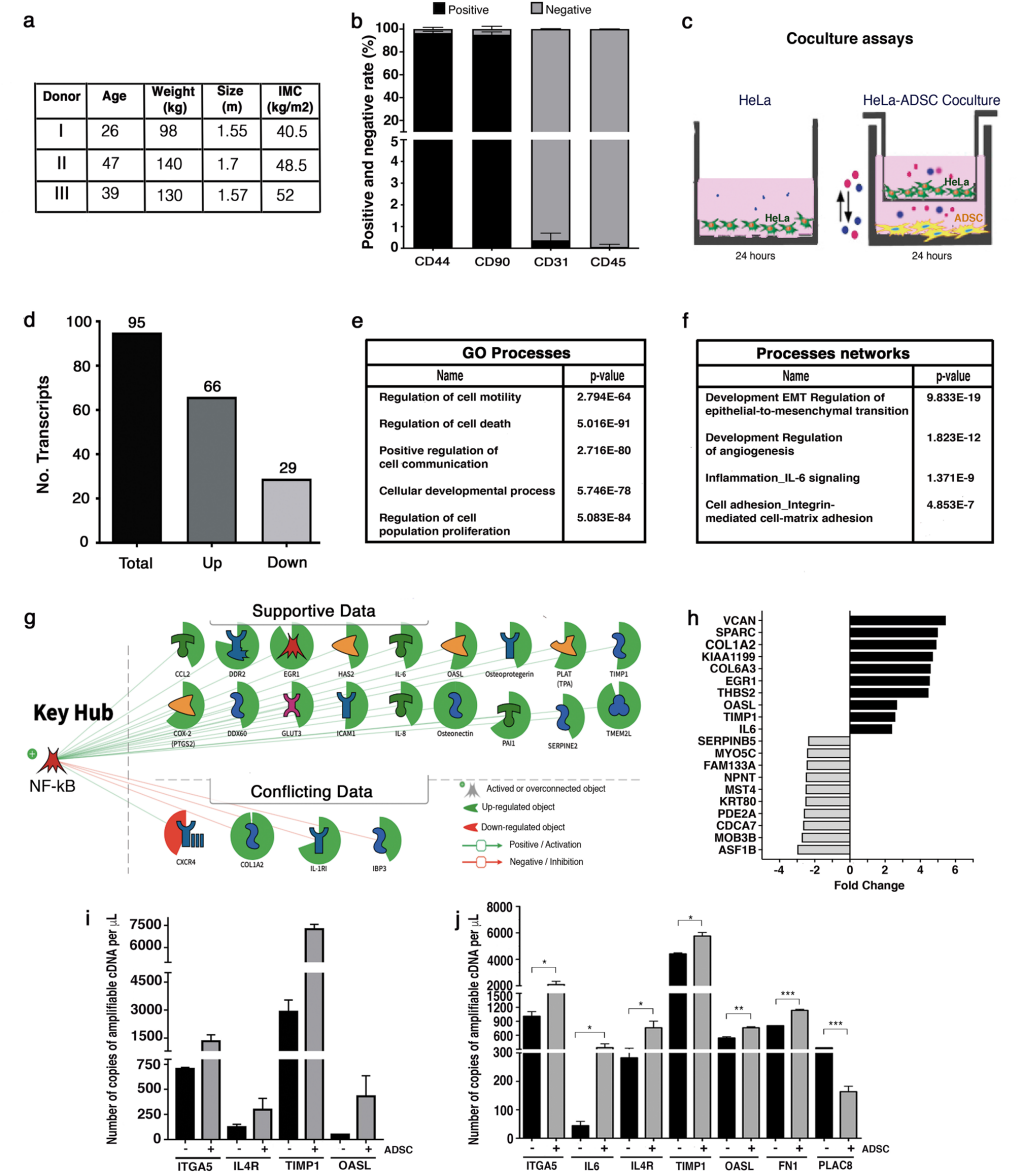
The coculture of HeLa/ADSC induces changes in the transcriptome of HeLa cells. (**a**) ADSCs were obtained from three donor patients. The table summarizes the main characteristics of the donor patients who underwent gastric bypass. (**b**) The purity of ADSCs in each patient was evaluated by flow cytometry analysis of cell surface markers including CD44, CD90, CD31 and CD45. The graph shows the percentage of positive and negative cells to each marker (n = 3 patients, 2 replicates, error bars = s.d.). (**c**) HeLa cells were cultured alone or in the presence of ADSCs by an indirect coculture system. The picture show that both cells lines are cultured in the same medium, but they are physically separated by a permeable membrane avoiding direct contact. (**d**) Bar chart shows the mRNAs altered in HeLa cells due to the presence of ADSCs. (**e**) The table shows the main molecular and cellular processes altered during coculture in HeLa. (**f**) The table shows the processes networks enriched during coculture in HeLa. (**g**) Schematic overview showing NF-kappa B as essential key hub driving gene expression, which was predicted to be activated in HeLa cells cocultured with ADSCs. (**h**) Top 20 differentially expressed genes in HeLa during coculture with ADSCs. (**i**–**j**). Quantification of gene expression by ddPCR showing the validation of mRNAs altered in HeLa by the presence of ADSCs obtained from patients (**j**) or ATCC (**k**). Graphs represent three biological replicates, and the error bars are s.d., *p < 0.05).

ADSCs were derived successfully from adipose tissue of 3 female cancer-free patients with morbid obesity (Fig. 1a). After a couple days of culture, ADSCs exhibited a stable spindle-shaped morphology (Supplementary Fig. 1). To further verify the identity and purity of ADSCs, we employed flow cytometry to examine the cell surface markers of three different cultures. We confirm that most of the cells express the appropriate positive ADSCs markers (CD44 and CD90) and very few cells express negative markers (CD31 and CD45). These results show that ADSCs exhibit the typical immunophenotype of ADSCs with expression rates of CD44 (96%), CD90 (95%), CD31 (0.36%) and CD45 (0.16%) (Fig. 1b). Taking together those results we verify that primary cultures are constituted by an enriched population of ADSCs.

### Coculture of HeLa/ADSCs induce changes in the transcriptome of HeLa cells

To assess whether ADSCs influence the behavior of CC cells, we cocultured HeLa cells with ADSCs obtained from patients. We employed an indirect coculture system where both ADSCs and CC cells are cultured under the same conditions but are physically separated by a permeable membrane thus avoiding direct contact but allowing cell communication (Fig. 1c). To evaluate the effect of ADSCs on the behavior of CC cells, we first evaluated the transcriptome of HeLa cells with the aim to identify the main processes and molecules affected by the presence or absence of ADSCs.

Transcriptome analysis revealed that HeLa cells cocultured with ADSCs have a total of 95 differential expressed (DE) RNAs (fold change > 2 or < − 2, p-value < 0.05 and FDR < 0.1) of which 66 transcripts were consistently upregulated and 29 were downregulated (Fig. 1d).

To elucidate the effects of ADSCs on the behavior of cervical cancer cells, we performed a network enrichment analysis using Ingenuity Pathway Analysis (IPA), Metacore and Key pathway Advisor (KPA). As shown in Fig. 1e, the interaction of ADSCs with HeLa cells disrupts the expression of genes involved in cellular processes, including cell motility, cell death, cell communication, developmental process, and proliferation. In addition, we found specific processes networks enriched in our data, these include the epithelial-mesenchymal transition, angiogenesis, inflammation mediated by interleukin-6 (IL6) signaling and cell adhesion (Fig. 1f), these networks represent a recognized larger group of interactions and/or pathways. Remarkably, EMT was the most enriched process in our data, those results suggest that ADSCs may influence HeLa cells to induce EMT, a biological process that allows epithelial cells to lose their polarity and undergo biochemical changes to acquire migratory and invasive properties. Besides, the most deregulated top genes are mainly associated with migration, cell adhesion, invasion, and metastasis (Fig. 1h).

In addition, KPA and Metacore predict that the activity of NF-kappa B is altered in HeLa cells cocultured with ADSCs and could be an essential regulatory hub, which drives differential expression changes (Fig. 1 g). Interestingly, it has been shown that NF- kappa B is essential for both the induction and maintenance of EMT in many kinds of cancer cells^41–44^. Taken together those results suggest that HeLa cells may acquire migration abilities probably associated to the induction of EMT.

To validate sequencing data we employed both ADSC-derived from Mexican patients and an ADSC cell line obtained from ATCC. We performed Digital PCR to quantify the expression levels of differentially expressed mRNAs. Our data showed that all transcripts analyzed (ITGA5, IL4R, TIMP1 and OASL) were successfully validated in both ADSCs obtained from Mexican patients and ADSCs provided from ATCC. Figure 1i–j shows that all mRNAs shift its expression levels in HeLa cells due to the presence of both patient-derived ADSCs or ADSC obtained from ATCC. We further analyze the expression of three more transcripts (IL6, FN1, and PLAC8) using ADSC obtained from ATCC and observed that all mRNAs were well validated (Fig. 1j).

**Figure 2:**
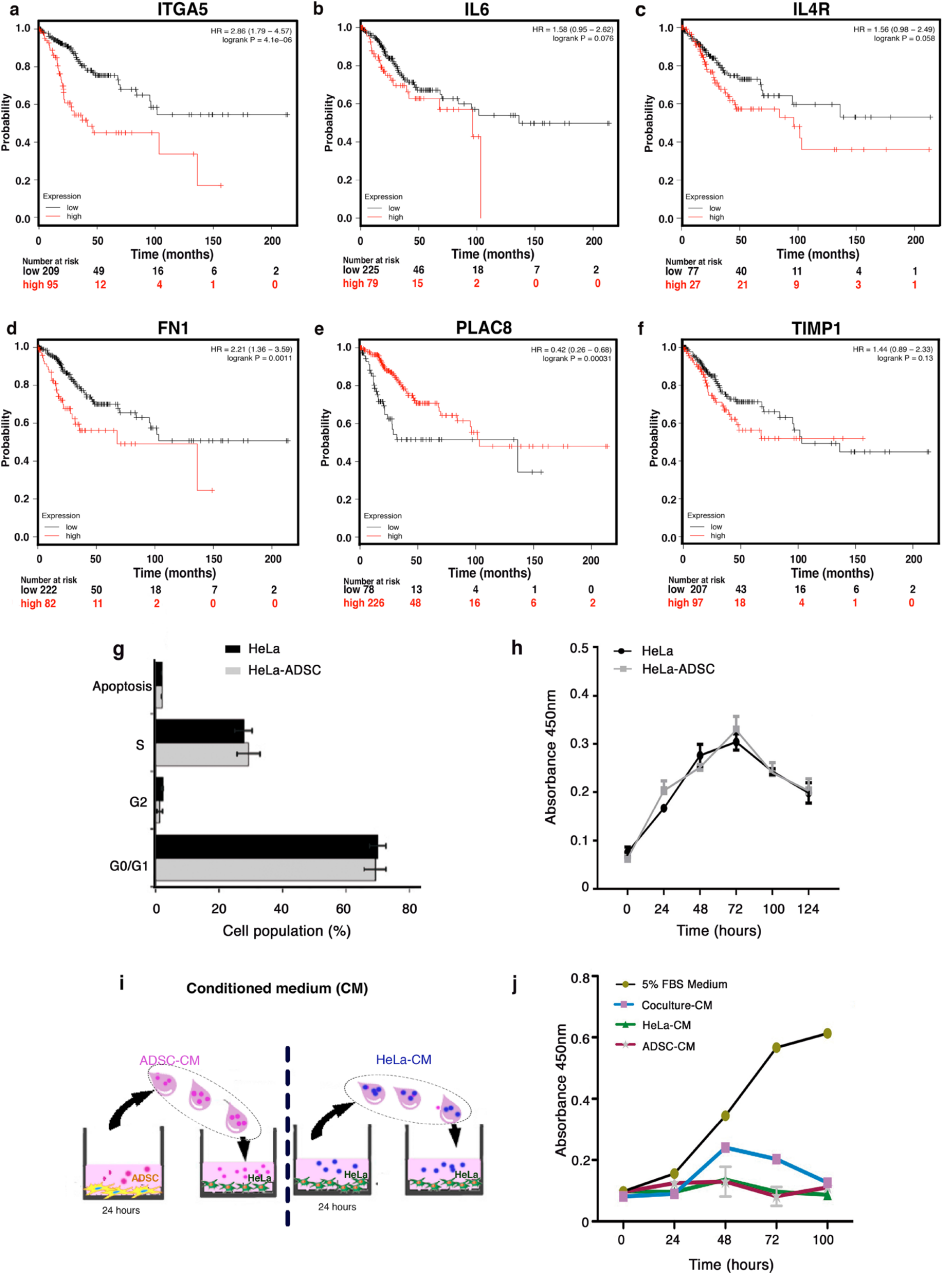
Deregulated genes altered by the presence of ADSC exhibit clinical significance in cervical cancer patients. (**a**–**f**) Kaplan Meyer curves comparing the overall survival of 304 cervical squamous cell carcinomas with low versus high expression of altered genes (ITGA5, IL6, IL4R, FN1, PLAC8, TIMP1). Data were obtained from a public database: KM Plotter. The “p” values are shown in each of the graphs. ADSCs do not alter the proliferative capacity of HeLa cells. (**g**) Cell cycle analysis of HeLa cells cocultured in the presence of ADSCs compared to HeLa control cells. (n = 3, the error bars are s.e.m) The graph shows no significant changes in any of the phases of the cell cycle. (**h**) Proliferation assay of HeLa control vs HeLa cells cocultured at different times. (**i**) The picture shows the method to obtain the conditioned medium of ADSCs (ADSC-CM) or the conditioned medium of HeLa cells (HeLa CM). ADSCs or HeLa cells were cultured in serum-free DMEM for 24 h and then the conditioned medium was employed to culture CC cells. (**j**) Proliferation assay of HeLa cells cultured with different conditioned media (CM) at different times. (n = 3, the error bars are S.D., but no significant changes were shown).

We performed a survival analysis with six DE RNAs using expression data from 304 patients with CC. Kaplan–Meier curves show the association between gene expression and cancer survival (Fig. 2a–f), data indicate that patients with high expression of ITGA5 and FN1 exhibit lower survival (Fig. 2a,d), while the low expression of PLAC8 is associated with shorter survival (Fig. 2e). However, the expression of IL6, ILR4, TIMP1 was not significantly associated with cancer survival and may possibly be associated with another biological process altered by ADSC (Fig. 2b,c,f). Taken together, these data suggest that ADSCs could increase the malignant phenotype of CC.

### Effect of ADSCs on the proliferation of CC

The expression of genes involved in proliferation was altered during the coculture of HeLa/ADSCs; however, the cell cycle analysis showed no changes (Fig. 2g). We also did not find any difference in the number of HeLa cells respect to HeLa cells cocultured with ADSCs in a proliferation assay with MTS during 124 h (Fig. 2h). In addition, we tested the ability of tumor cells to proliferate in the presence of different conditioned media obtained from ADSCs, HeLa cells or HeLa-ADSCs (Fig. 2i–j). HeLa cells cultured with DMEM at 5% FBS were used as a positive control (Fig. 2j). There was no significant change in the proliferation of HeLa cells treated with conditioned medium. Overall, these findings demonstrate that ADSCs have no influence on the proliferation and death of tumor cells.

### ADSCs promote the migration and invasion of CC

Bioinformatic analysis predicts cell migration to be altered in HeLa cells cultured in the presence of ADSCs (Fig. 1e). To assess whether ADSCs influence the migration or invasion abilities of CC, we used transwells and evaluated the behavior of HeLa, CaSki and SiHa cells cultured in the presence of some chemoattractants, including ADSCs, conditioned medium obtained from the coculture of HeLa/ADSCs, conditioned medium of ADSCs and fibroblasts (NIH3T3). Conditioned mediums were obtained under serum free conditions after 24 hr of the coculture of CC cells in the presence of ADSCs (Coculture CM) or the culture of ADSCs alone (ADSCs CM). As a positive control of migration, cells were stimulated with DMEM supplemented with 10% FBS and as a negative control we employed DMEM without FBS.

**Figure 3:**
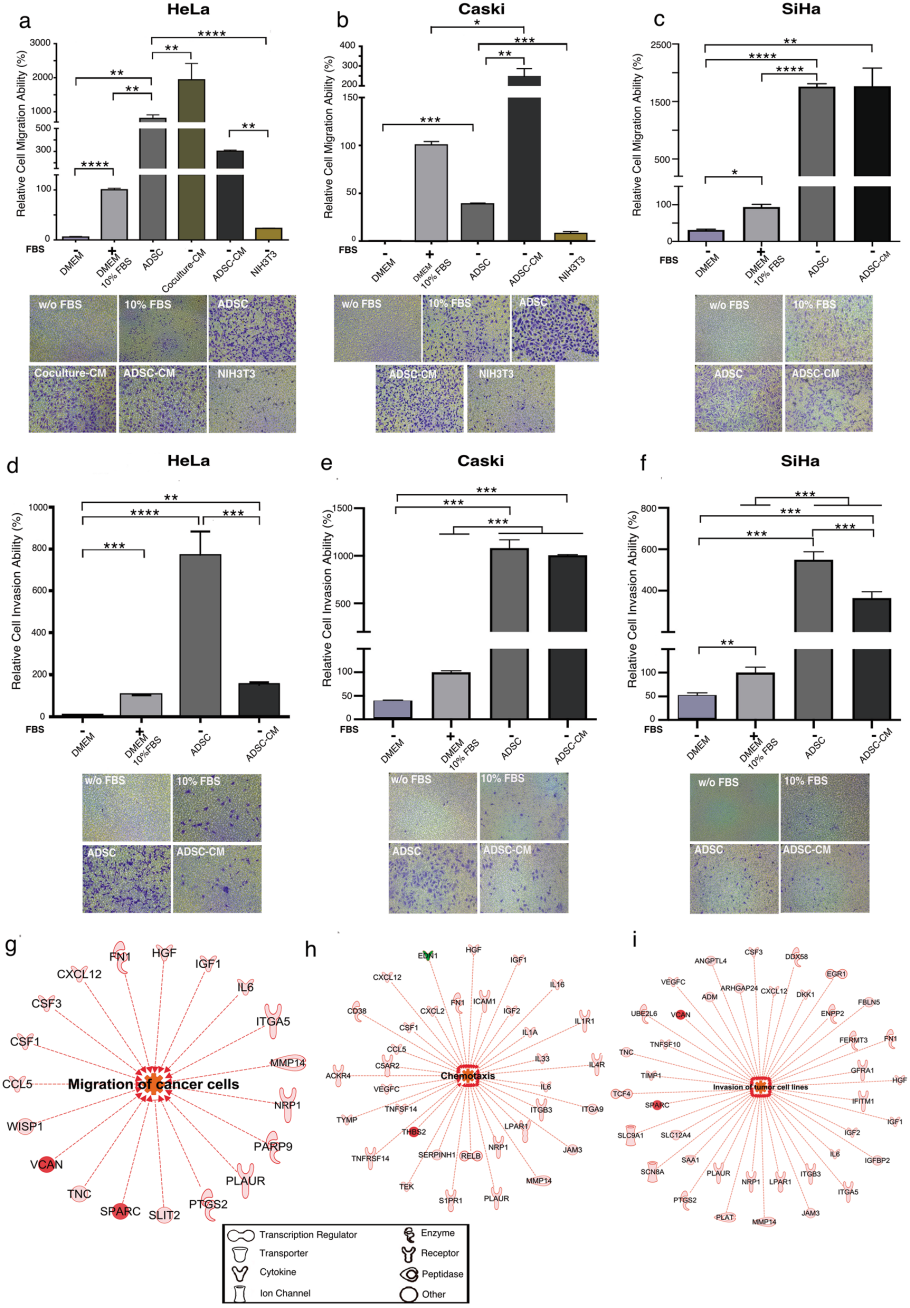
ADSCs influence the migration and invasion of cervical cancer cells. Graph shows the migration ability of HeLa (**a**), CaSki (**b**) and SiHa (**c**) cells cultured without serum and exposed to different chemoattractants including ADSCs, conditioned medium of coculture (Coculture-CM), conditioned medium of ADSCs (ADSC-CM) and NIH3T3 cells. As a control, CC cells were also cultured with DMEM supplemented with 10% FBS or without FBS. The graph shows the relative percentage of the migration capacity of HeLa, CaSki and SiHa cells after 12 h (**a**–**c**). The graph represents three biological replicates, error bars are s.d and *p < 0.05. Pictures show a representative image of the migratory CC cells in each condition. (**d**–**f**) Figures show the relative percentage of invading HeLa, CaSki and SiHa cells after 12 h of exposure to various chemoattractants. Figures show a representative image of invading cells in each condition. The graph represents three biological replicates, error bars are s.d and *p < 0.05. IPA analysis showing that the main altered transcripts in HeLa cells cultured in presence of ADSCs are involved in migration (**g**), chemotaxis (**h**), and invasion (**i**). The networks show differentially expressed genes regulated by each signaling pathway. The red color indicates the overexpression of the transcripts.

Our results showed that ADSCs promote a drastic increment in the migration ability of HeLa, CaSki and SiHa cells compared to unstimulated cells (Fig. 3a–c). Strikingly, ADSCs acted as a powerful chemoattractant since HeLa and Siha cells reached 807% and 1758% more migration abilities compared to cells stimulated with 10% FBS (Fig. 3a,c). Interestingly, HeLa cells exhibit very little migration ability when stimulated with fibroblast (NIH3T3) (Fig. 3a). These results highlight the biological significance of ADSCs in improving the migration of cancer cells. To test whether the factors secreted by ADSCs influence the migration of CC cells, we test the migration ability of CC cells stimulated with conditioned medium of ADSCs. Our results showed that conditioned medium of ADSCs increase the migration of HeLa (300%), CaSki (245%) and SiHa (1885%) cells when compared with cells stimulated with 10% FBS. These results indicate that CC cell lines expose to the ADSCs or the conditioned medium of ADSCs exhibit a dramatic increment in the migration ability of CC.

Because invasion allows cells to spread toward distant sites and colonize tissues, we determined the influence of ADSCs on the invasion of CC. We performed invasion assays using Matrigel-coated Boyden chambers, as shown in Fig. 3d–f, the invasion capacity of HeLa, CaSki and SiHa cells drastically increases 767%, 1,080% and 549% respectively when they are chemoattracted by ADSCs in contrast to cells stimulated with 10% FBS. Consistently, the conditioned medium of ADSCs also increase the invasion of all CC cells (Fig. 3d–f).

Taken together, these data indicate that ADSCs induce profound changes in cell migration and invasion, thus conferring advantages to CC. Interestingly, an IPA analysis showed that different regulated genes were positively associated with the invasion and migration process in cancer cells: VCAN, SPARC, IL6, MMP14, IL4R, ICAM, TIMP1 and IGF2 (Fig. 3g–i).

**Figure 4:**
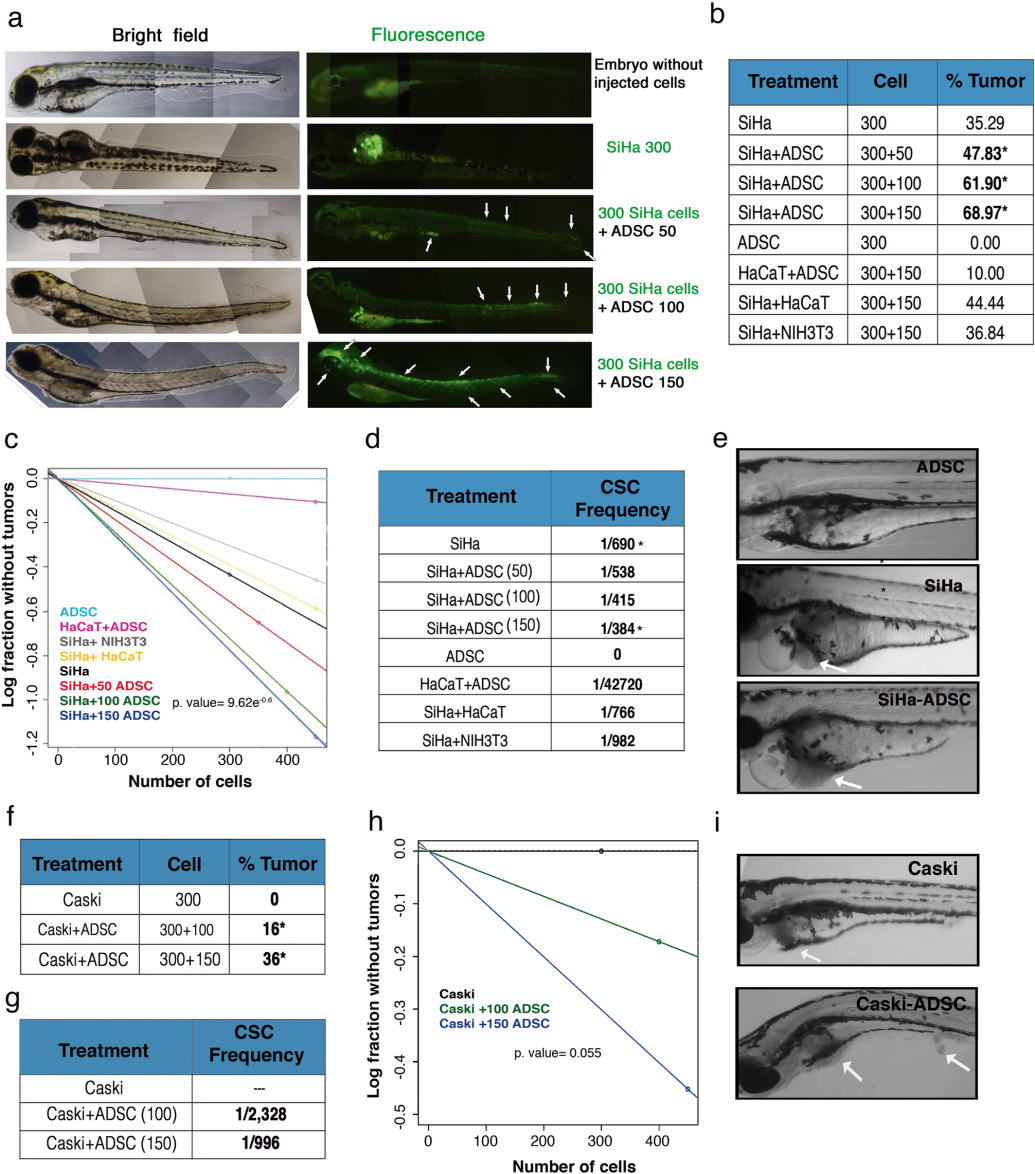
ADSC increases the CSC population, migration and invasion in an in vivo model. (**a**) Images show that the migration capacity of SiHa cells increases proportionally with respect to the amount of ADSCs inoculated in zebrafish embryos after 12 h. SiHa cells are shown in green due to they were transfected with a plasmid harboring a GFP gene. The images show a gradual increase in the migration and invasion of cancer cells from the yolk to the tail of embryos due to the presence of ADSCs. The white arrows show the migration areas in the embryo. (**b**) The table shows the number of cells inoculated in zebrafish embryos and the proportion of tumors formed in each condition. SiHa, SiHa + ADSC, or control cells: ADSC, HaCaT + ADSC, SiHa + HaCaT and SiHa + NIH3T3 were inoculated into zebrafish embryos, and the tumors were monitored every day for 5 days. (**c**) The graph depicts the frequency of CSC in each condition representing the number of cells injected with respect to the logarithmic fraction of animals without tumors. (**d**) The table shows the frequency of CSC calculated based on the ELDA software. (**e**) The images show tumors developed during 5 days in zebrafish embryos inoculated with ADSC, SiHa or SiHa + ADSC cells. Each experiment was repeated at least three times. (**f**) The table shows the number of cells inoculated in zebrafish embryos and the proportion of tumors formed in embryos injected with CaSki cells or CaSki + ADSCs cells. (**g**) The table shows the frequency of CSC calculated based on the ELDA software. (**h**) The graph depicts the frequency of CSC in each condition representing the number of Caski cells injected with respect to the logarithmic fraction of animals without tumors. (**i**) The images show tumors developed during 5 days in zebrafish embryos inoculated with CaSki or CaSki + ADSC cells.

To confirm the influence of ADSCs on the migration or invasion ability of CC cell lines, we conducted in vivo experiments using zebrafish embryos (*Danio rerio*). Those experiments were performed in SiHa cell line, due to it has a higher tumorigenic ability than HeLa (data not shown). SiHa cells were transfected with a plasmid carrying a GFP gen (Fig. 4a) or were stained with the PKH26-GL dye (Supplementary Fig. 2). A total of 300 SiHa cells expressing GFP or labeled with a dye in the presence or absence of ADSCs were injected into the zebrafish embryonic yolk sacs at 48 hpf. The number of inoculated cells did not affect embryo viability during a 7-day trial (data not shown). Migration and invasion were evaluated at 12 h after the injection. Embryos injected only with SiHa displayed few fluorescent sporadic cells throughout the embryo; in contrast, SiHa cells inoculated with ADSCs exhibited higher migration observed throughout the body, eyes and tail of the embryo. In some cases, we observed cell invasion and subsequent tail metastasis. These data suggest that the presence of ADSCs promotes the migration and invasion ability of CC cells (Fig. 4a and Supplementary Fig. 2).

### ADSCs promote tumor growth

Subsequently, we evaluated whether ADSC could contribute to CC progression; thus, we performed coinjections of 300 SiHa cells with different dilutions of ADSC cells (50, 100 or 150) and monitored tumor growth. In addition, tumor formation was also monitored in coinjected embryos with the following controls: ADSC + HaCaT, SiHa + HaCaT and SiHa + NIH3T3 (Fig. 4b). As shown in Fig. 4b, the coinjection of SiHa cells with different numbers of ADSCs promoted an increase in the number of embryos with a tumor; notably, this increase was dependent on the number of ADSCs injected. The results show that SiHa cells produced tumors in 35% embryos, while SiHa-ADSCs could develop tumors in 69% embryos. As expected, ADSCs alone did not develop any tumor, even when these cells were inoculated in higher amounts. The coinjection of ADSC/HaCaT only developed tumors in 10% embryos, while SiHa/HaCat or SiHa/NIH3T3 exhibited the same tumorigenic potential as SiHa cells injected alone.

In addition, we calculated the frequency of cancer stem cells (CSCs) in each condition through an ELDA analysis. Figure 4c and d shows an increment in the CSC proportion observed in tumors formed by SiHa-ADSC with 1 CSC per every 384 cells; these tumors have approximately 55.6% more CSCs than in the tumors derived from SiHa alone. As expected, tumors derived from HaCaT + ADSC, SiHa + HaCaT or SiHa + NIH3T3 exhibit lower numbers of CSCs than in the tumors derived from SiHa. Interestingly, SiHa cells coinjected with ADSC produced larger tumor nodules compared with tumors generated only by SiHa cells (Fig. 4e).

The relevance of ADSCs in the tumorigenesis, is further supported by another CC cell line, in which we observed that CaSki cells by themselves are unable to form tumors while cells inoculated with ADSCs can produce tumors in 36% embryos (Fig. 4f,i). Furthermore, we observed that the number of CSCs increased in CaSki cells inoculated with higher amounts of ADSCs (Fig. 4g). As shown in Fig. 4h, CaSki cells inoculated with bigger amounts of ADSCs had 1 CSC per every 996, while cells injected with lower quantity of ADSCs only had 1 CSC per 2,328 cells. These results indicate that the presence of ADSCs improves the tumorigenic potential of CC cells and increases the frequency of CSCs.

### ADSC induces the activation of the NF-kappa B pathway in CC cells

**Figure 5:**
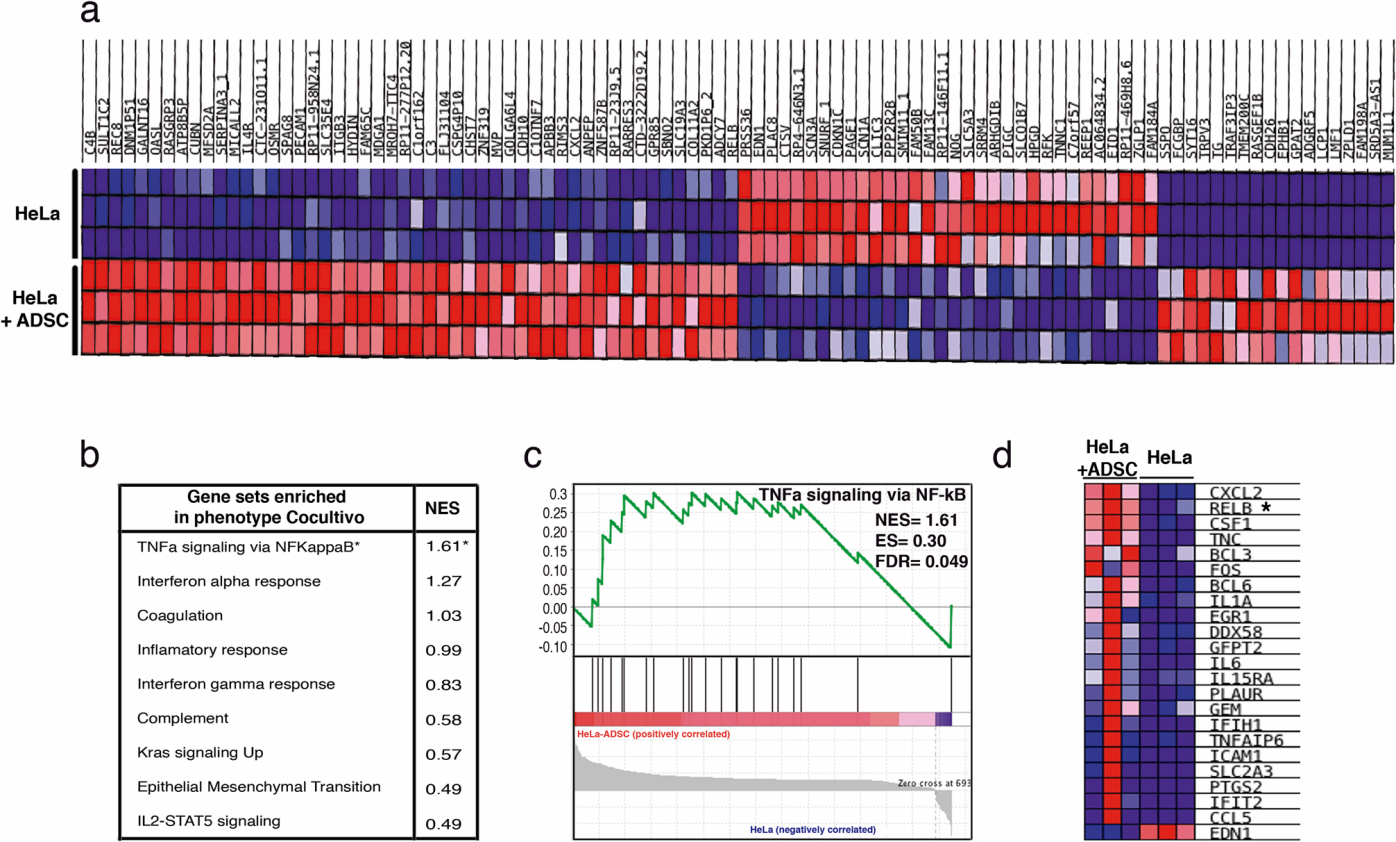
The NF-Kappa B pathway is the most activated pathway during the coculture of HeLa-ADSC. (**a**) Heat map shows the main differentially expressed genes in the HeLa cell line cultured in the presence or absence of ADSC. The expression values are represented as colors, where the range of colors (red, pink, light blue, dark blue) represents the range of expression values (high, moderate, low, lowest). (**b**) Table shows the main phenotypes enriched in HeLa cells due to the presence of ADSCs obtained from a gene set enrichment analysis (GSEA). For each of the phenotypes, the normalized enrichment score (NES) is indicated. (**c**) Analysis of GSEA showing significant enrichment (NES = 1.61 and an FDR = 0.049) of the TNFα-NF-kappa B signaling pathway in cervical cancer cells due to the presence of ADSCs. (**d**) The heat map shows the subset of enriched genes involved in the TNFα-NF-kappa B pathway, where most deregulated genes are involved in the noncanonical NF-Kappa B pathway, such as RELB.

Interestingly, the data analysis with IPA software suggests that most deregulated genes culminate in NF-kappa B pathway activation during the coculture of HeLa/ADSCs. A Gene Set Enrichment Analysis" (GSEA) confirmed that NF-kappa B signaling was enriched (p-value = 0, FDR = 0.049 and NES = 1.61) in HeLa cells cultured in the presence of ADSCs (Fig. 5b and c). Figure 5a shows a heat Map exhibiting differentially expressed genes with high significance. Among these genes, we found RelB, a player in noncanonical NF-kB signaling, which is highly expressed in HeLa/ADSCs. The GSEA showed 23 DE RNAs associated with the NF-kappa B pathway, including CXCL2, RELB, BCL3, FOS, BCL6, IL1A, and IL6 (Fig. 5c,d).

**Figure 6:**
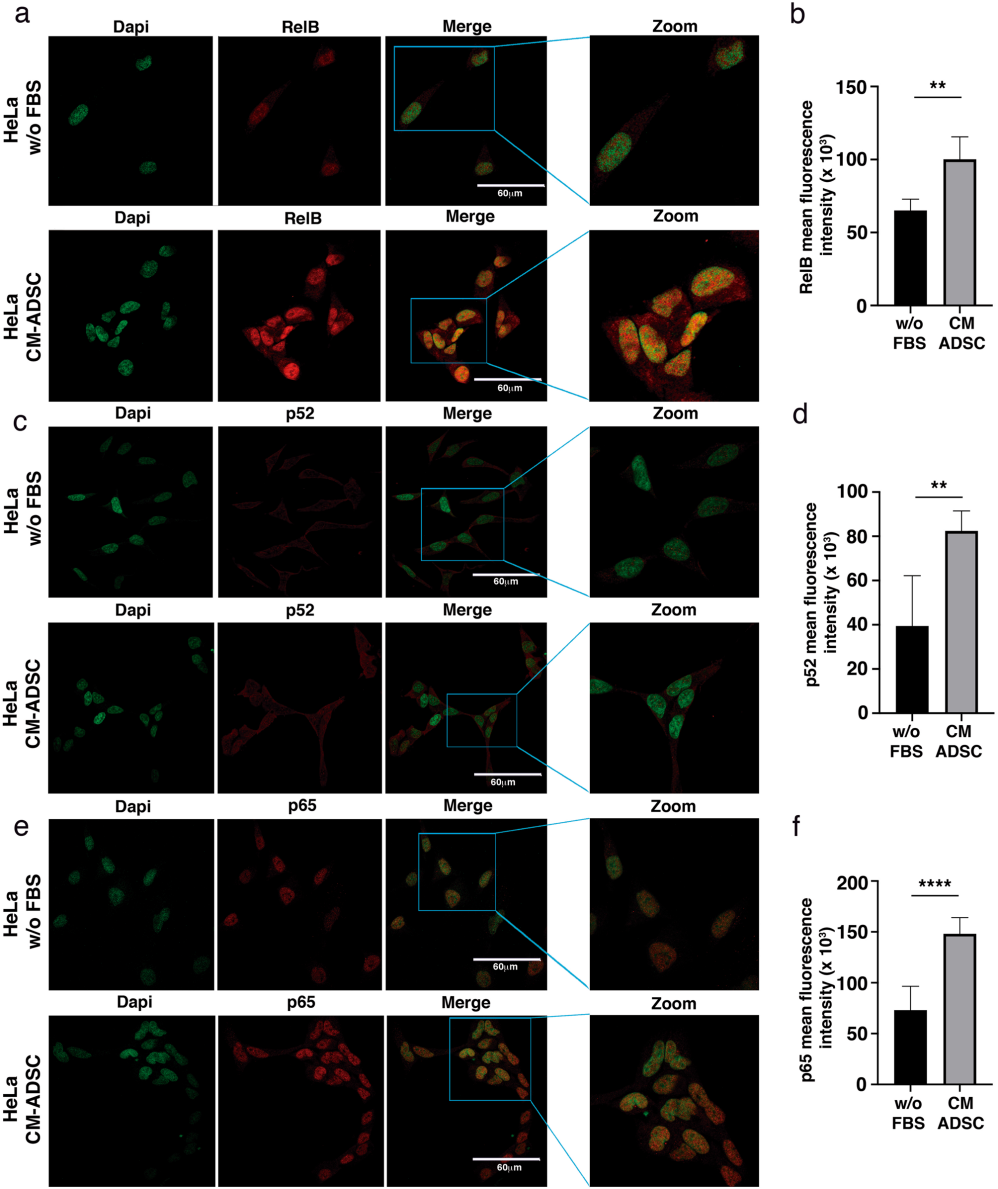
ADSCs induce the expression of the NF-kappa B molecules in CC cells. (**a**–**f**) The photographs show the expression of transcription factors of the NF-Kappa B family including RelB (**a**), p52 (**c**) and p65 (**e**) obtained by immunofluorescence of HeLa cells cultured with serum-free DMEM medium or ADSC-CM (free of serum) for 24 h. The images were taken under a confocal microscope. The scale bar = 60 μm. The cell nuclei were contrasted with DAPI. Each experiment was repeated at least three times and to quantify the expression levels of Relb (**b**), p52 (**d**), and p65 (**f**), the intensity of fluorescence was quantified using ImageJ. Graph represents three biological replicates, error bars are s.d. and ****p < 0.0001 and **p < 0.01.

To validate and determine which NF-kappa B molecules are involved in the CC regulation mediated by ADSC, we performed immunofluorescence analysis of key NF-kappa B proteins (Fig. 6). As shown in Fig. 6a, immunofluorescence analyses showed that the conditioned medium of ADSCs induced an increase in RelB expression (Fig. 6a,b) as well as its nuclear translocation in HeLa cells. In addition, we observed a slight increase in p52 phosphorylation in HeLa cells cultured with the conditioned medium of ADSCs (Fig. 6c,d). Interestingly, the levels of p65, a canonical NF-kappa B player, were also increased, thus implying crosstalk between canonical and noncanonical NF-kappa B signaling (Fig. 6e–f). In fact, accumulating evidence suggests that RelB can regulate the expression of subunits of the canonical NF-kappa B pathway.

**Figure 7:**
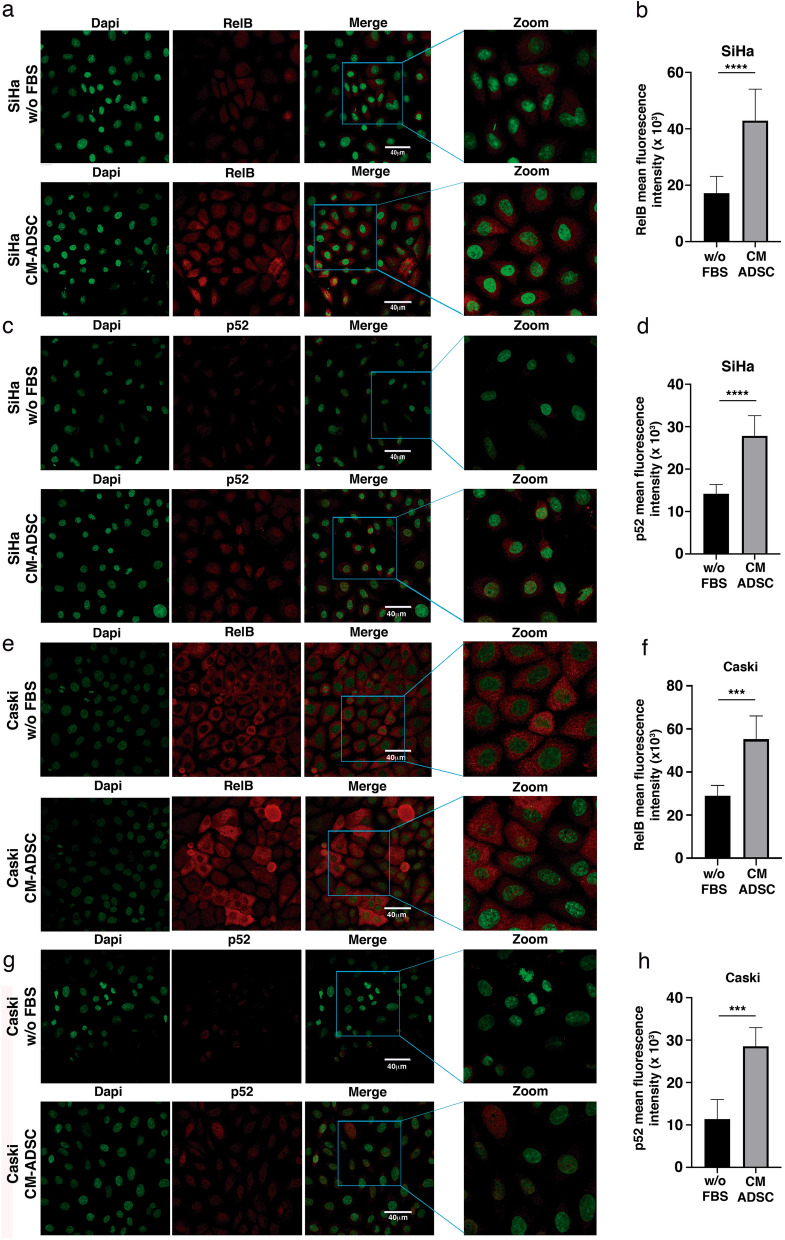
ADSC induces the activation of the NF-kappa B pathway in SiHa and CaSki cells. (**a**–**h**) The photographs show the expression of RelB and p52 obtained by immunofluorescence of SiHa (**a**–**c**) or CaSki (**e**–**g**) cells cultured with serum-free DMEM or ADSC-CM (free of serum) for 24 h. The images were taken under a confocal microscope. The scale bar = 40 μm. The cell nuclei were contrasted with DAPI. Each experiment was repeated at least three times. To quantify the expression levels, the intensity of fluorescence was quantified using Image J. Graphs show the mean fluorescence intensity of RelB (**b**), p52 (**d**) in SiHa and Relb (**f**) and p52 (**h**) in Caski cells. Graph represents three biological replicates, error bars are s.d. and ****p < 0.0001 and *** p < 0.001.

To verify the involvement of ADSCs in the regulation of non-canonical NF-kappa B molecules, we also analyze the expression of p52 and RelB in SiHa or CaSki cells cocultured with conditioned medium of ADSCs (Fig. 7a–h). We observe that the conditioned medium of ADSCs induced a notorious increment in the expression of both RelB (Fig. 7a,b) and p52 (Fig. 7c,d) in the SiHa cells. We also observed that some CaSki cells exhibit an increment in the expression of RelB when they are cultured with conditioned medium of ADSCs (Fig. 7e,f). Supporting previous results, we also demonstrated that the conditioned medium of ADSCs induces an increment in the phosphorylation of p52 in the CaSki cells (Fig. 7g,h). Taking together these results suggest that ADSCs could induce the activation of the noncanonical NF-kappa B pathway.

### ADSC induces a stem cell phenotype and EMT in CC cells

**Figure 8:**
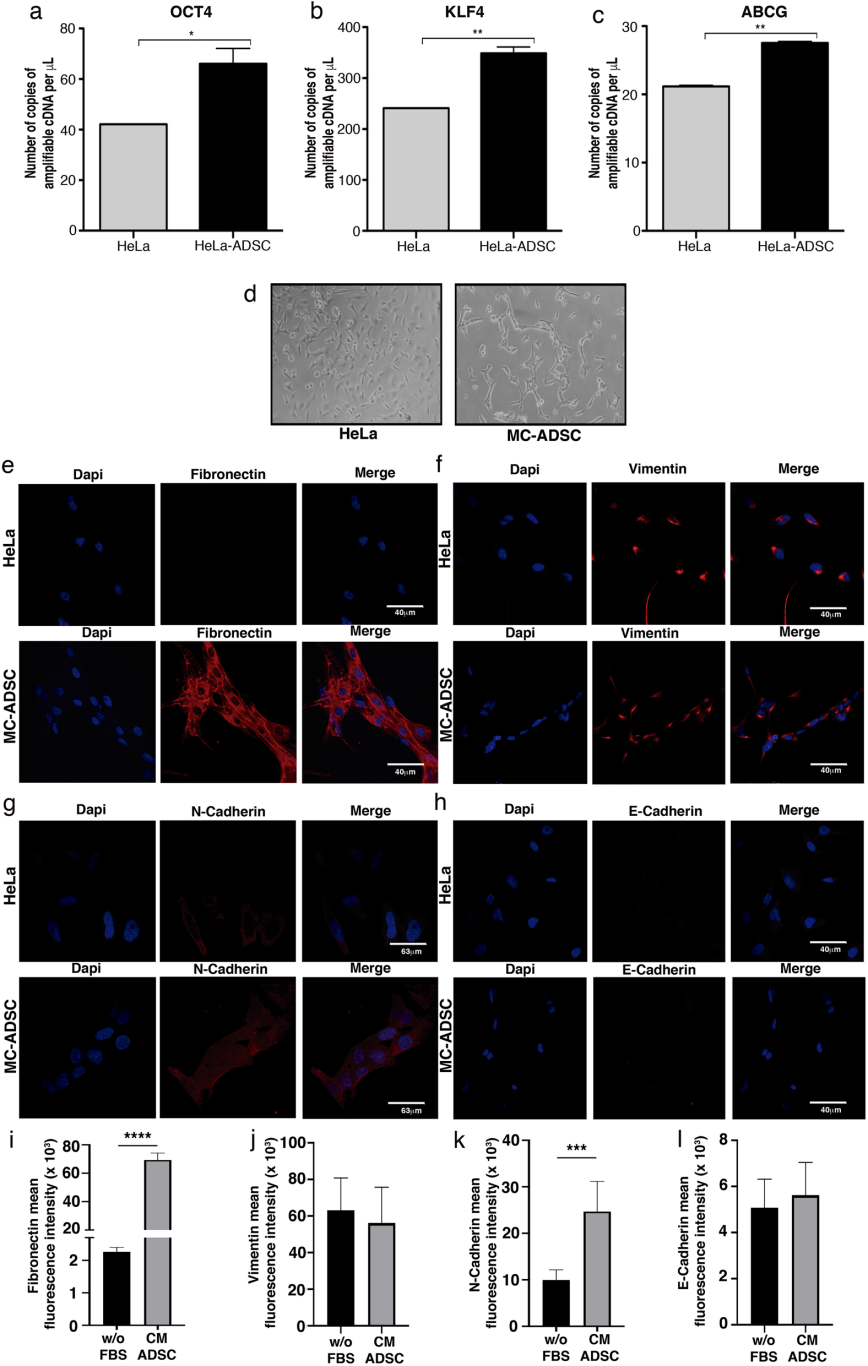
ADSC promotes a stem cell and EMT phenotype in HeLa cells. (**a**–**c**) Graphs show the expression level of pluripotency genes evaluated by ddPCR in HeLa control vs HeLa cells cocultured with ADSC. Pluripotency genes include OCT4 (**a**), KLF4 (**b**) and ABCG (**c**). The error bars represent the means ± standard deviation (SD). (**d**) Schematic representation shows that cervical cancer cells exhibit an EMT-like phenotype induced by the conditioned medium of ADSC. Expression of EMT markers including fibronectin (**e**), Vimentin (**f**), N-cadherin (**g**) and E-cadherin (**h**) analyzed by immunofluorescence of HeLa cells cultured with or without conditioned ADSC. EMT proteins were stained with Cy3-conjugated secondary antibody and nuclei were stained with 4′-6-Diamidino-2-phenolindole (DAPI). Images were taken in a confocal microscope using an × 40 oil lens. Photographs are representative of three independent experiments. (**i**–**j**) To quantify the expression levels of fibronectin (**i**), vimentin (**j**), N-cadherin (**k**) and E-cadherin (**l**), the intensity of fluorescence was quantified using Image J. Graph represents three biological replicates, error bars are s.d and ****p < 0.0001 and ***p < 0.001.

**Figure 9:**
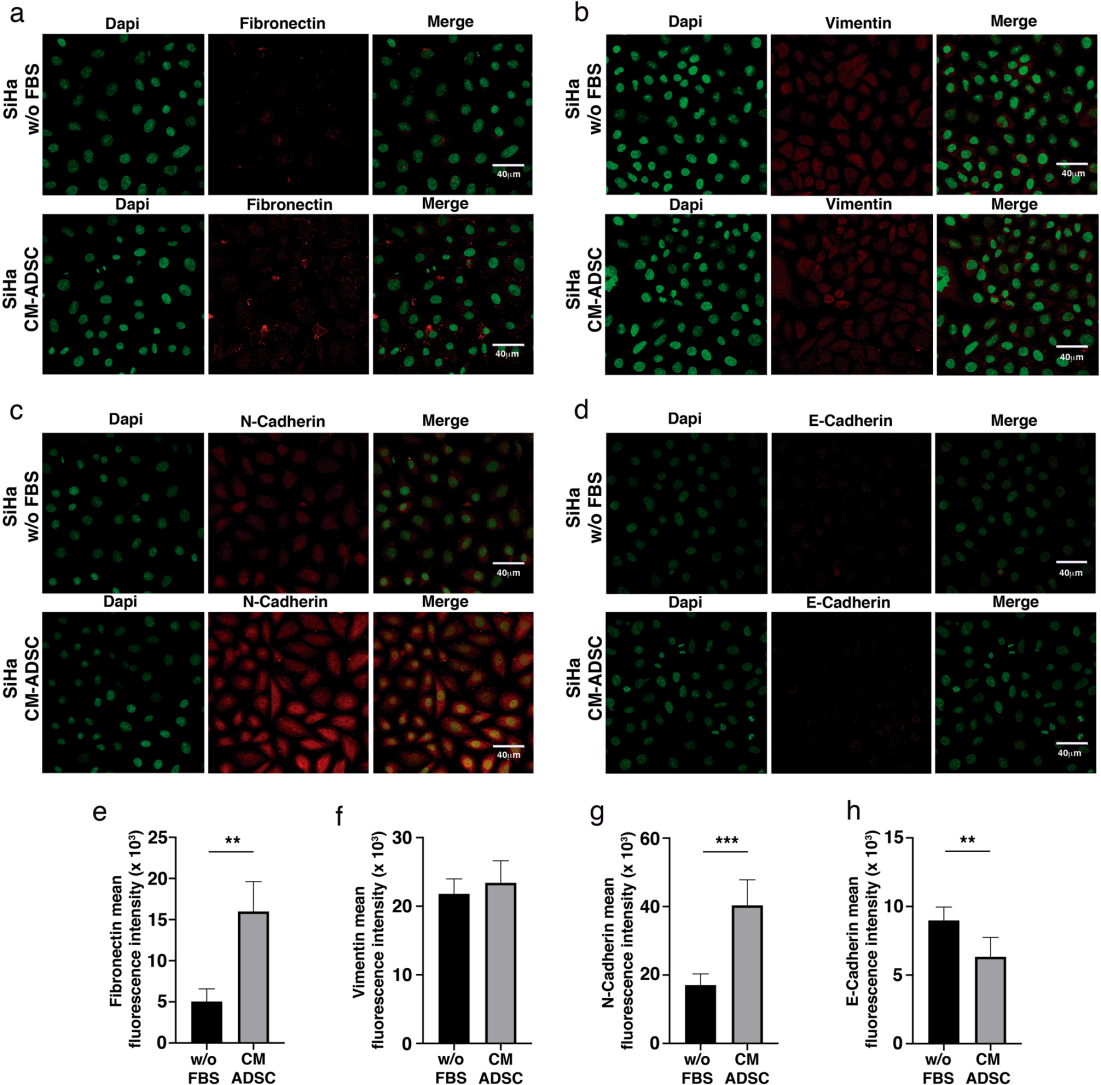
ADSC induce an EMT phenotype in SiHa cells. (**a**–**d**) Immunofluorescence analysis of EMT markers including fibronectin (**a**), Vimentin (**b**), N-cadherin (**c**) and E-cadherin (**d**) in SiHa cells cultured with or without conditioned medium of ADSC. EMT proteins were stained with Cy3-conjugated secondary antibody and nuclei were stained with 4′-6-Diamidino-2-phenolindole (DAPI). Images were taken in a confocal microscope using an × 40 oil lens. Photographs are representative of three independent experiments. To quantify the expression levels of fibronectin (**e**), vimentin (**f**), N-cadherin (**g**) and E-cadherin (**h**), the intensity of fluorescence was quantified using Image J. Graph represents three biological replicates, error bars are s.d. and **p < 0.01 and ***p < 0.001.

Compelling evidence suggests that NF-kappa B signaling participates in the regulation of stem cell-associated genes. Notably, RelB is overexpressed in the mesenchymal fraction of some tumor types, so we assessed whether ADSCs could increase stemness in HeLa cells. Our results showed that HeLa cells cultured in the presence of ADSCs exhibit increased expression of stem cell markers, such as OCT4, KLF4 and ABCG (Fig. 8a–c). These results are in agreement with the ELDA analysis, which calculated a higher frequency of CSCs in tumors grown by SiHa and CaSki cells inoculated in the presence of ADSCs (Fig. 4d,h). Remarkably, we observed that HeLa cells cultured in the conditioned medium of ADSCs exhibit a distinctive morphonology similar to a mesenchymal phenotype with elongated shape and loss of cell contact (Fig. 8d). This feature is consistent with the bioinformatic analysis inferring that the EMT program is altered in HeLa/ADSCs. To assess the role of ADSCs in the regulation of EMT markers, we evaluated the protein levels of fibronectin, N-cadherin, Vimentin and E-cadherin in HeLa and SiHa cell lines. The results showed an evident increment in the mesenchymal markers, HeLa cells exposed to conditioned medium of ADSCs had higher levels of Fibronectin (Fig. 8e,i) and N-cadherin (Fig. 8g,k), however, we could not find any significant change in the mesenchymal marker Vimentin (Fig. 8f,j) and E-cadherin (Fig. 8h,l), a well-known epithelial marker, perhaps due to its low expression in this cell line. Corroborating these results, we found that SiHa cells exposed to conditioned medium of ADSCs exhibited a drastic increment in Fibronectin (Fig. 9a,e) and N-cadherin (Fig. 9c,g), two key hallmark molecules of EMT. We also find a slight decrement in E-cadherin (Fig. 9d,h), a well-known epithelial marker. Collectively, these findings support the role of ADSCs in the metastasis of CC by promoting the migration and invasion of cancer cells through the induction of an EMT program.

### ADSC modulates angiogenesis in CC tumors

**Figure 10:**
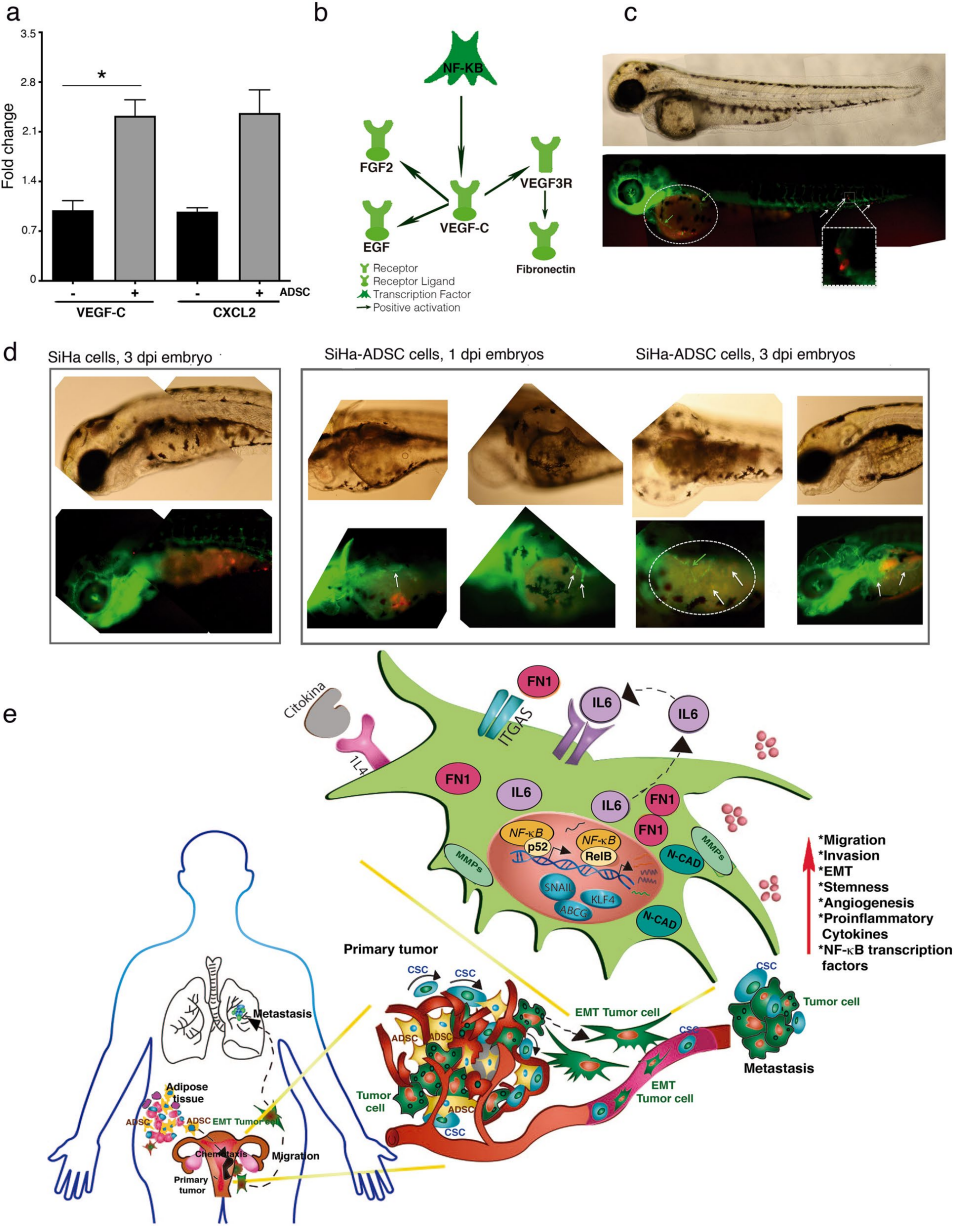
ADSC modulate angiogenesis in CC tumors. (**a**) Graph shows the fold increase of DE mRNAs involved in angiogenesis obtained from HeLa/ADSC RNAseq data. Angiogenesis-related mRNAs shown are VEGF-C, and CXCL2. (**b**) Representative network of KPA analysis showing that NF-Kappa B/VEGF-C activates the signaling of angiogenic factors in HeLa cells cocultured in the presence of ADSC. (**c**) Representative photograph of the infiltration of SiHa cells in the blood vessels of fli1a embryos: EGFP after 12 h post-injection is shown. The SiHa cells are observed in red color and blood vessels in green. (**d**) Representative figure showing the formation of new blood vessels in the yolk of embryos from day 1 dpi and 3 dpi of SiHa cells vs SiHa-ADSC. (**e**) The illustration built with the experimental data obtained, indicates that the ADSCs migrate from the adipose tissue to CC tumor and there, they increase the malignant phenotype of the CC cells and promoting metastasis. The ADSC potentiate the malignant phenotype of cervical cancer cells by increasing the non-canonical NF-Kappa B pathway, causing an increase in the expression of chemokines, transcription factors, metalloproteases, integrins, etc., which contribute to the increased migration and invasion capacity of cancer cells. In addition, ADSC activate the EMT process mainly due to the increase of Fibronectin in CC cells. Finally, ADSCs induce the angiogenic potential that plays an important role in tumor progression. It should be mentioned that all these phenotypic changes could be given by the activation of the NF-Kappa B pathway.

Our analysis of sequencing showed an increase in molecules with mitogenic and pro-angiogenic activities during HeLa-ADSC coculture. These molecules included a chemokine CXCL2 as well as the ligand VEGF-C, a proangiogenic factor actively released by the tumor cells for the formation of new vessels (Fig. 10a). Studies have shown that these factors are regulated by the NF-Kappa B pathway^45,46^. Bioinformatics analysis predicted that compared to HeLa cells, HeLa-ADSCs possess a higher angiogenic potential. Through a KPA analysis, we validate that the NF-Kappa B pathway promotes the activation of VEGF-C and that this protein can bind and activate to FGF2 and EGF ligands as well as VEGFR-3 receptor, which activates fibronectin (Fig. 10b).

To test whether ADSCs influence the ability of cervical tumors to establish angiogenesis, we evaluated the in vivo effects of ADSCs in the vasculature development of transgenic zebrafish embryos (fli1a: EGFP) inoculated with SiHa/ADSC or SiHa cells only. These embryos express EGFP specifically in the vascular vessels, thus allowing the monitoring of new blood vessels. Supporting the above results, we also observed a clear migration of SiHa cells into the bloodstream mainly in embryos injected with SiHa/ADSCs (Fig. 10c). We monitored vascularization from 1 up to 3 days and observed that SiHa cells failed to form new blood vessels; however, SiHa cells inoculated in the presence of ADSCs formed vascularized tumors (Fig. 10d).

## Discussion

Cancer, cardiovascular diseases and diabetes mellitus account for more than 70% of deaths worldwide^47^. In addition, cervical cancer remains the fourth cause of cancer incidence (13.1) and cancer death (6.9) in women worldwide^48^.

Obesity is an important risk factor for cancer, and it has been associated with a decrease of 5 to 20 years in the survival of cancer patients. According to data from the OCED, Mexico ranks second in the worldwide prevalence of obesity (BMI ≥ 30 kg/m^2^)^47,49,50^. It is increasingly clear that obese women exhibit a higher risk of developing CC compared to women with normal BMI^51–53^. Although the characteristic cell of adipose tissue is the adipocyte, this is not the only cell type present in this tissue, other cells such as preadipocytes, macrophages, neutrophils, lymphocytes and endothelial cells are harbored in the adipose tissue and have also been linked to obesity and cancer.

Studies linking obesity and cancer have focused on endocrine and metabolic repercussions that promote a generalized inflammatory process^54–56^, however, little is known about the specific molecular mechanism by which obesity increases the risk of cancer.

In this project, we studied ADSCs which are also harbored in the adipose tissue and are essential players in tissue development and regeneration^57^. ADSCs are found in abundant quantities in the adipose tissue, it has been reported that ADSCs represents up to 30% of total cells contained in this tissue^58,59^. Notably, recent studies have provided evidence that the number of ADSCs exceeds the frequency of marrow-derived mesenchymal stem cells (BMSC) found in the medullary stroma^60^. Approximately, 1 ml of adipose tissue obtained by liposuction contains about 6 × 10^5^ to 2 × 10^6^ ADSCs^61^, this represents at least 500 times more cells than the number of stem cells obtained from bone marrow^62–64^. Taken together these data highlight adipose tissue as the richest source of multipotent stem cells, possibly outperforming any other source in the body.

Remarkably, recent evidence has shown that patients with obesity exhibit higher systemic mobility of ADSC than healthy patients and interestingly, this mobilization is even higher in cancer patients^65^, which supports the incorporation of ADSCs into tumors.

Accumulating evidence has demonstrated that these cells possess the ability to migrate toward tumor sites by chemoattraction^11,19^. The role of ADSCs in cancer has not been clearly elucidated, and there are some reports that evidence the malignant behavior of ADSCs allowing rapid growth of tumors. In contrast, some studies have shown that ADSCs exhibit antitumoral potential. The role of ADSCs in cervical cancer is still unknown,thus, it is necessary to determine the effect of ADSCs on CC^66,67^. In this study, we demonstrated for the first time that ADSCs increase the malignant phenotype of cervical cancer cell lines, such as HeLa, SiHa and CaSki cells.

We used an indirect coculture system in which both tumor cells and ADSCs were physically separated through a permeable membrane and cultured in the same microenvironment, thus allowing paracrine signaling. This method has been used to study the effect of ADSCs on other tumors, such as squamous cell carcinoma^21^, breast cancer^17,68^, melanoma^69^, lung and colorectal cancer^22^.

Our results show that the presence of ADSCs (CD90^+^/CD44^+^/CD31^−^/CD45^−^) alters the transcriptome of CC cells. Data obtained from RNA-seq revealed that 95 RNAs were differentially expressed in HeLa cells cocultured in the presence of ADSCs derived from patients. Gene expression changes were validated by digital PCR, and we found that both ADSCs obtained from patients or from ATCC exhibit similar gene expression patterns, thus corroborating this phenomenon. Interestingly, most deregulated genes, such as IL6, RelB, RelA, Plac8, ITGA5, CXCL12 and FN1, among other cytokines and growth factors, have been involved in tumor growth and progression. Preisner and colleagues used qRT-PCR to analyze the expression of 229 tumor-promoting genes in melanoma cells cocultured with ADSCs and found similar cytokines and growth factors, such as IL6, CXCL12, VEGF, and HGF^69^.

Interestingly, validated genes (ITGA5, IL6, IL4R, FN1, TIMP1, and PLAC8) are highly relevant in CC because their expression level is directly related to CC patient survival. These genes have also been relevant in other tumor types. For example, ITGA5, an integrin that promotes tumor invasion^70^, has been correlated with lower survival of lung cancer patients^71^, and it also functions as a receptor for FN1^72,73^, which was also upregulated in our coculture. IL4R induces a prometastatic phenotype in epithelial tumors.

Integral analysis with IPA, KPA, and GSEA suggests that ADSCs modified cellular processes, such as migration, invasion, angiogenesis and proliferation. Accumulating evidence has demonstrated that ADSCs can also influence the proliferation of cancer cells; however, we could not observe any influence of ADSCs on the proliferation ability of HeLa cells. The migration, invasion, and angiogenesis were corroborated using in vitro and in vivo strategies. Supporting our results, various studies have demonstrated the effect of ADSCs on the migration and invasion of melanoma, breast, prostate and pancreatic cancer. Strikingly, we found many DE RNAs related to migration and invasion, such as VCAN, SPARC, MMP14, ITGA5, PLAUR, NRP1, IL-6, CXCL2, and HGF. Notably, we also observed drastic morphological changes in HeLa and SiHa cells cocultured with ADSCs, thus conferring migratory abilities perhaps by inducing epithelial to mesenchymal transition.

Our results also show that ADSCs modify the behavior of CC cells, thus inducing rapid tumor growth and metastasis. Mechanistically, little is known about how ADSCs facilitate tumor development. Recent evidence has shown that ADCS provoke an increase in IL6 levels, which induces the phosphorylation of JAK2/STAT3^16^, resulting in proliferation, invasion and tumor growth in prostate and endometrial cancers. Strikingly, we also found that ADSCs induce a dramatic increase in IL6/STATs and an increase in noncanonical NF-kappa B signaling in HeLa and SiHa cells. These results are consistent with a recent study showing that mesenchymal stem cells promote colorectal cancer progression through NF-kappa B activation^74^. Our results show for the first time that the NF-kappa B pathway in CC is activated due to the presence of ADSCs. The bioinformatic analysis detects 23 NF-kappa B-related genes and suggests that RelB, a member of the noncanonical NF-kappa B pathway, is the main growth factor involved in the regulation of NF-kappa B signaling. We also found that the expression of p52 and p65 is induced by ADSCs, suggesting that both canonical and noncanonical signaling pathways are activated in CC.

Notably, ADSCs induced a rapid expansion of CSCs in zebrafish inoculated with SiHa and CaSki in the presence of ADSCs. It is well established that CSC expansion rapidly induces tumor growth and development, which further suggests that an increase in the number of CSCs is responsible for the highly invasive and metastatic abilities conferred by ADSCs. One way by which CC cells may acquire stemness is due to the constitutive activation of NF-Kappa B. Recent evidence has clearly established that NF-kappa B dramatically expands the number of stem cells and increases the clonogenicity and self-renewal abilities of CSCs^25,75,76^. Our results indicate that ADSCs induce the overexpression of stem cell markers such as OCT4, KLF4, and ABCG in HeLa cells. In addition, we found that ADSCs promote HeLa and SiHa cells to undergo EMT, thereby acquiring mesenchymal features verified by the high expression of fibronectin, n-cadherin, and vimentin. In addition, it is increasingly clear that EMT induces the acquisition and maintenance of stem cell-like features. These results are in accordance with recent research showing that glial tumor and lung cancer cells are able to undergo EMT and acquire mesenchymal features when they are cultured in the presence of conditioned medium obtained from ADSCs.

Finally, we also observed that DE RNAs, including VEGFC, CXCL2, VEGF3R, FGF2 and EGF, are also involved in angiogenesis. We performed in vivo assays and consistently observed that ADSCs are able to induce the growth and formation of new blood vessels that could supply nutrients and oxygen to the tumors. Interestingly, Lin CS et al. demonstrated that ADSCs migrate toward prostatic tumor sites and increase tumor vascularity mediated by FGF2^19^. In addition, Dexheimer et al. demonstrated that the conditioned medium of the coculture of ADSCs and squamous cell carcinoma induces tube formation in human umbilical vein endothelial cells (HUVECs), thus corroborating the proangiogenic role of ADSCs^21^.

In conclusion, our results demonstrate that ADSCs affect multiple features that contribute to malignancy of cervical cancer cells such as gene expression, migration, invasion, angiogenesis, and stemness. In spite of the contribution of ADSCs into the promotion of those features, ADSCs have no effect in the proliferation of cervical cancer cells (Fig. 10e). Interestingly, the effects of ADSCs found in patient-derived ADSCs were also validated in an ADSC cell line obtained from ATCC.

It is important to say that ADSCs could promote features that contribute to malignancy of cervical cancer through the NF-kB signaling pathway and the induction of EMT (Fig. 10e). In addition, we observed that mRNAs altered by the presence of ADSCs exhibit clinical significance since they are associated with shorter survival of cervical cancer patients. Although the clinical relevance of the ADSC cells remains incomplete, our results give guidelines to the search for new molecules that can reduce the mortality rates of cervical cancer obese patients. We believe that much work needs to be done to fully elucidate the exact mechanism involved in the contribution of ADSCs in the malignant phenotype of cancer cells.

Furthermore, due to the malignant role of ADSCs in cancer, it is necessary to evaluate the safety of ADSC-based therapies in order to avoid the co-localization of those cells with cancer cells which could promote tumor growth.

## Supplementary information

Supplementary Information.
