# Supplementary material for: Adipose-derived mesenchymal stem cells promote the malignant phenotype of cervical cancer
Source: Sci Rep. 2020 Aug 26;10:14205. doi: 10.1038/s41598-020-69907-x (PMC7450089; doi:10.1038/s41598-020-69907-x)
Supplement: Supplementary file 1 — Supplementary Information. [file 41598_2020_69907_MOESM1_ESM.pdf]

# Adipose-derived mesenchymal stem cells promote the malignant phenotype of cervical cancer

**Rosario Castro-Oropeza, Karla Vazquez-Santillan, Claudia Díaz-Gastelum, Jorge Melendez-Zajgla, Cecilia Zampedri, Eduardo Ferat-Osorio, Arturo Rodríguez-González, Lourdes Arriaga-Pizano, and Vilma Maldonado.**

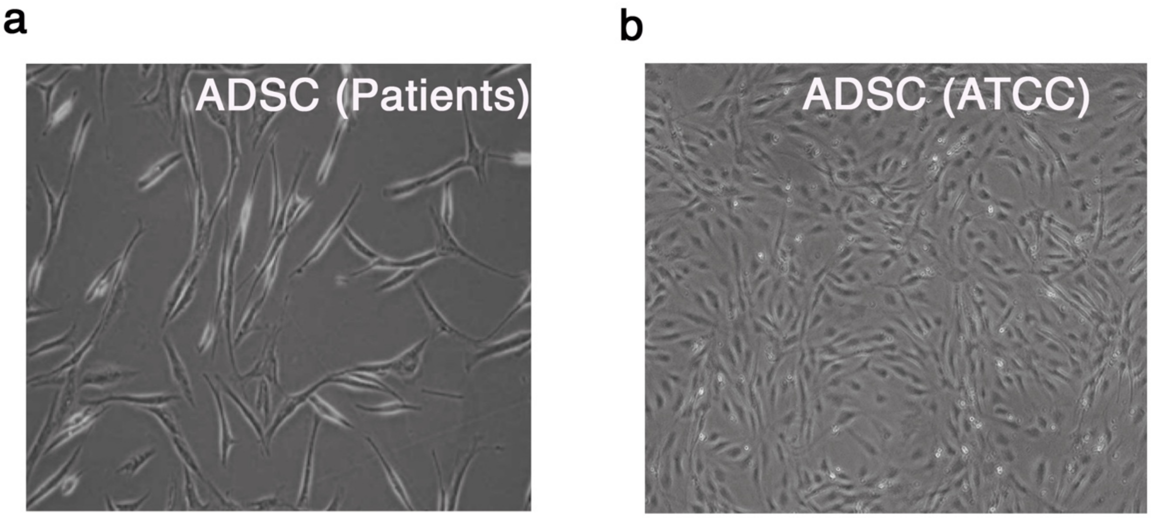


**Supplementary Figure 1**

**Isolation of ADSCs obtained from patients undergoing bypass gastric.** (a-b) Picture shows the ADSCs isolated from adipose tissue of Mexican patients (a) or obtained from ATCC (b).

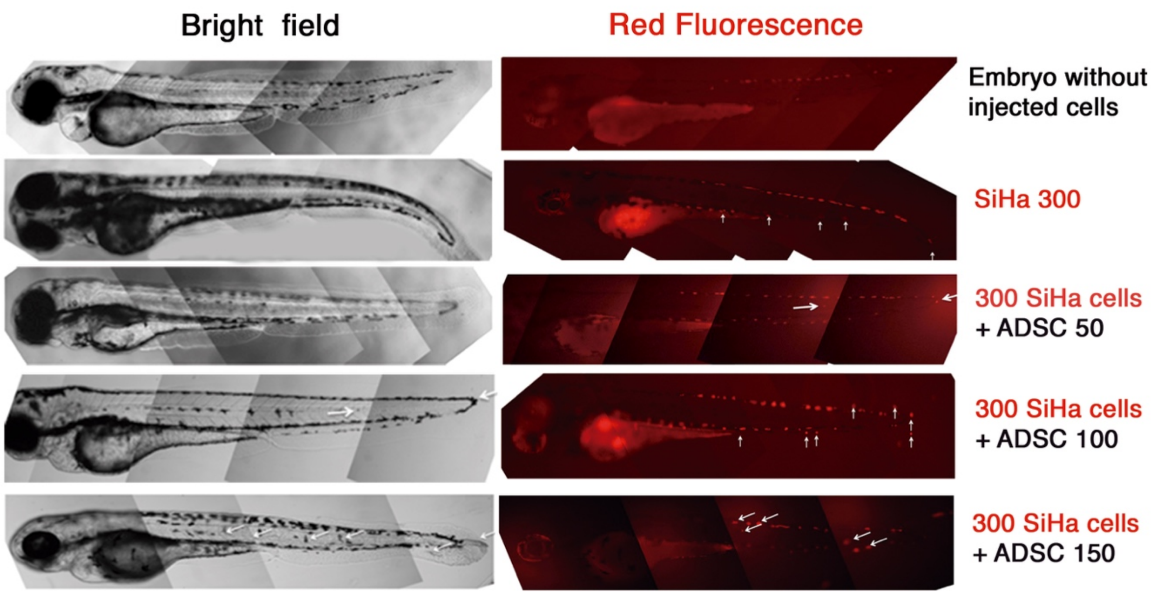


**Supplementary Figure 2**

**ADSC increases the migration and invasion of CC cells in an in vivo model.** (a) Images show that the migration capacity of SiHa cells increases proportionally with respect to the amount of ADSCs inoculated in zebrafish embryos after 12 hrs. SiHa cells are shown in red due to the staining with the PKH26 dye. The images show a gradual increase in the migration and invasion of cancer cells from the yolk to the tail of embryos due to the presence of ADSCs. The white arrows show the migration areas in the embryo.
